# Racial Climate on Willingness to Participate in Clinical Research

**DOI:** 10.1007/s40615-025-02530-6

**Published:** 2025-07-07

**Authors:** Sanaz Dabiri, Rema Raman, Linda B. Cottler, Catherine W. Striley, Jevay Grooms

**Affiliations:** 1 Sol Price Schaeffer Center &, Alzheimer’s Therapeutic Research Institute, University of Southern California, Los Angeles, CA USA; 2 Alzheimer’s Therapeutic Research Institute, University of Southern California, Los Angeles, CA USA; 3 Department of Epidemiology, College of Public Health and Health Professions and College of Medicine, University of Florida, Gainesville, FL USA; 4 Department of Economics, Howard University, Washington, DC USA

**Keywords:** Research participation, Recruitment, Racism, Underserved communities

## Abstract

**Objective:**

In the USA, there are striking racial and ethnic disparities in health outcomes particularly among African American individuals, compared to White individuals. Despite these disparities, recruiting African American communities for clinical research remains challenging. Using the socio-ecological model as a framework, this project examined whether the racial climate of the geographical area-including police shootings, neighborhood vulnerabilities, and individual trust in research institutions, is significantly associated with willingness to participate in clinical research.

**Methods:**

We analyzed data from the University of Florida HealthStreet registry, assessing community perceptions of clinical research participation. Data was aligned with Fatal Encounters police shootings and the Social Vulnerability Index by county and zip code.

**Results:**

Participants had an average age of 43.01 years (SD = 16.32) with an average education of 13.04 years (SD = 2.35). The majority were female (64%) and identified as Black/African American (56.2%). Multiple logistic regression results showed that higher rates of police shootings were significantly associated with lower willingness to participate in studies involving taking medicine (*b* =  − 0.124, se = 0.055, *p* < 0.05), providing samples for genetic studies (*b* =  − 0.001, se = 0.000, *p* < 0.05), and use of medical equipment (*b* =  − 0.021, se = 0.001, *p* < 0.001) among African American adults compared to White participants. Higher social vulnerability was associated with a lower willingness to participate in clinical research.

**Discussion:**

Socioecological factors’ contribution to clinical research participation among African American communities is poorly understood. Our findings highlight the need to address these factors to improve community engagement and reduce the diversity gap in clinical research.

**Supplementary Information:**

The online version contains supplementary material available at https://doi.org/10.1007/s40615-025-02530-6.

## Introduction

Despite the absence of a biological basis, the terms race and ethnicity are social constructs primarily influenced by physical characteristics that impact one’s access to power and social and economic resources [1] on community, national, and global scales. Consequently, populations that belong to racial and ethnic minoritized groups bear a disproportionate burden of adverse health outcomes [2]. For instance, the prevalence of Alzheimer’s disease and other dementias (AD/ADRD) among older adults is twice as high in African American/Black individuals compared to White individuals [3]. Similarly, African American/Black individuals are more likely to have cardiovascular risk factors and develop cardiovascular diseases compared to White individuals [4]. Despite an increased risk for adverse health outcomes, the underrepresentation of African American/Black individuals in clinical trials are well-documented [5, 6]. Clinical trial participants are often non-Hispanic White individuals, married or have a partner with a higher socioeconomic status [7]. This underrepresentation of minoritized communities limits the findings’ generalizability and availability of meaningful treatment for minoritized populations. Therefore, understanding the relationship between racial climate and clinical research participation is essential for addressing disparities in healthcare access and health outcomes. Considering the USA’s unique racialized social systems perspective [8], this study applies the socioecological framework to examine how the racial climate within geographical locations relates to African American/Black individuals’ decisions to participate in clinical research.

Contrary to the misconception of disinterest, a literature review encompassing 70,000 participants across 20 health research studies revealed that African American/Black and Hispanic individuals exhibited a similar willingness to participate in clinical trials as their non-Hispanic White counterparts [7]. However, Ofstedal and Weir [9] showed that African American/Black participants were less likely to consent to biological measurements, such as blood or saliva sampling, body mass index, blood pressure, and follow-up engagement that gathered data on medication, physical health, and psychological issues. During debriefing, these individuals most frequently cited mistrust of research as the reason for their decline [9]. Prior research has shown that perceived discrimination is significantly linked to medical distrust [10], while time constraints, marginalization, and higher opportunity costs [11] may further hinder participation. This distrust in healthcare is not an isolated sentiment; it reflects a broader skepticism toward institutions [12] where racial minoritized communities have faced a long history of discrimination and inequitable treatment [13]. For many African American/Black individuals, healthcare institutions are viewed as an extension of the same authority structures seen in government and law enforcement. Overall, the underrepresentation of African American/Black adults in AD/ADRD clinical research persists, largely due to factors such as mistrust and distrust of medical institutions, historical injustices, and socioeconomic barriers [10]. Such factors are closely tied to the racial climate within a given society.

### Framework

**Fig. 1:**
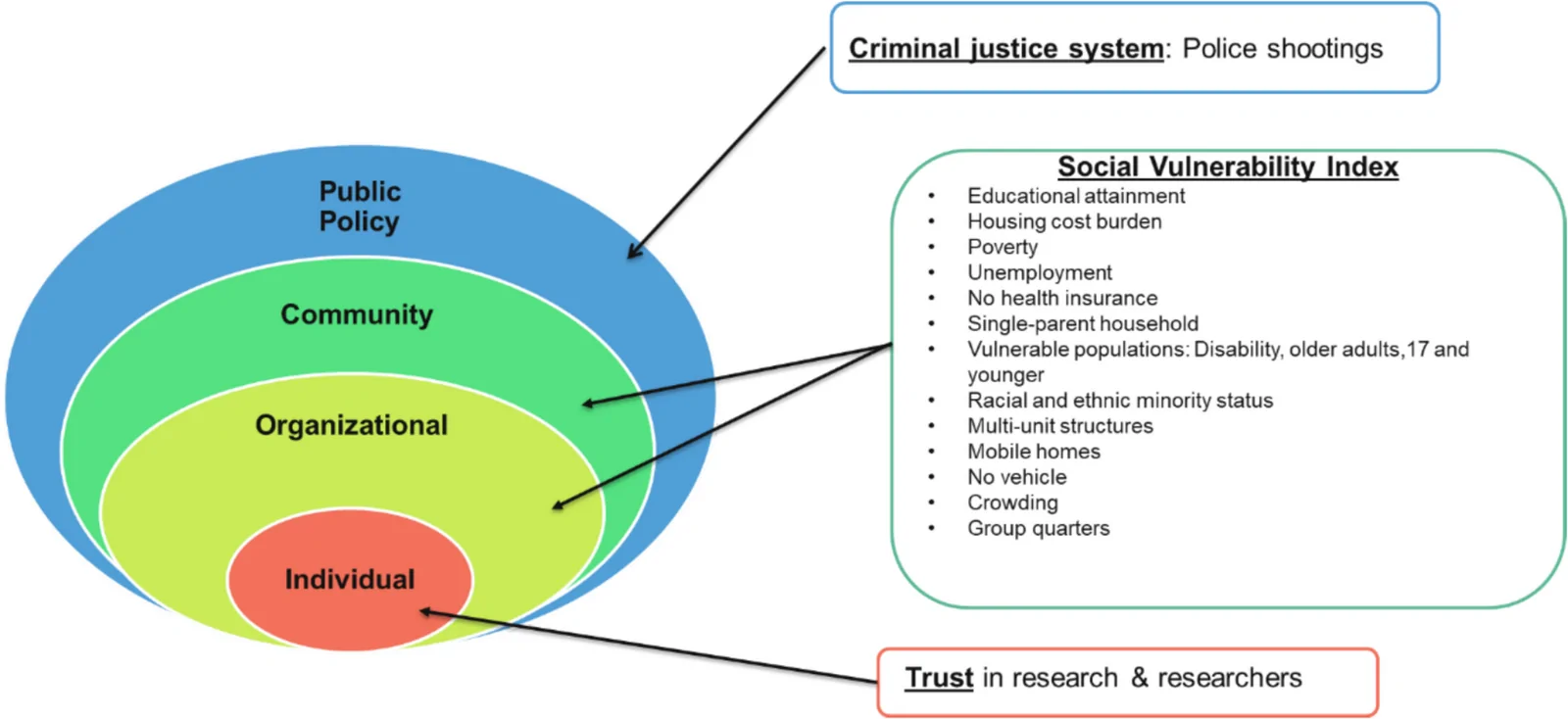
Socioecological model of an individual’s willingness to participate in clinical research given the racial climate

We conceptualized racial climate using the multilevel socioecological model (Fig. 1). Racial climate is intertwined with social determinants of health. It involves several factors, including demographic composition, education, housing discrimination in the neighborhood, community resources, political representation, racial disparities in policing, sentencing and prison population, availability of healthcare facilities and insurance coverage, and disparities in subjective well-being. Income inequality is negatively associated with health through the perception of relative deprivation and gradual deterioration of trust and social cohesion. Income inequality is also associated with lower-quality public education and health care [14]. Since racial and ethnic disparities in income and wealth are linked to health outcomes, examining a broader perspective on socioeconomic status is necessary to acknowledge historical patterns of racialized residential segregation with lower quality of life among African American/Black individuals compared to White individuals [1]. These intertwined systemic inequities illustrate how healthcare distrust is part of a broader experience of institutional inequity.

### Police Shootings

In addition to the inequities in healthcare, education, and wealth, shared experiences of fatal police shootings (e.g., killings of Eric Garner, Tamir Rice, George Floyd, and Breonna Taylor) is another form of oppression that African American/Black individuals endure. Police brutality encompasses both deliberate and accidental actions by law enforcement officers that degrade individuals, involving psychological coercion, neglect, verbal aggression, and instances of physical or sexual harm. African American/Black individuals are more likely to be stopped and arrested by the police and are more than three times more likely to endure injuries compared to White individuals [15–18].

These experiences with law enforcement, where African American/Black individuals frequently encounter harm and discrimination, are symptomatic of the same structural racism that affects healthcare access and treatment quality. A national survey of US urban adults found that individuals with experiences of police brutality have higher levels of medical mistrust, independent of their socioeconomic status, healthcare access, and health status [15]. Fatal police shooting, as a highly visible example of structural racism in the USA [19], contributes to a profound and justified distrust in institutions broadly, including medical institutions**.**

African American/Black individuals who experience such traumatic events are also more likely to report hypervigilance, depressed mood, and generalized anxiety [19]. In this heightened state of vigilance, individuals remain alert to potential discrimination and other dangers [20] often perceiving their surroundings as less trustworthy and unsafe-even after the traumatic experience has passed [21].

This distrust is not solely a product of contemporary events. African American/Black individuals have long faced racial discrimination both inside and outside of the healthcare settings, contributing to enduring skepticism toward clinical research and medical institutions [13]. The legacy of the Tuskegee Syphilis Study continues to resonate in African American/Black communities’, reinforcing fears of exploitation and distrust in clinical research and the healthcare system [22–26]. For example, a community-based participatory research exploring breast cancer treatment perceptions among African American/Black individuals found that participants frequently expressed distrust, invoking the idea of being treated like “guinea pigs” and referencing the Tuskegee study as an example of historical exploitation [26]. These collective experiences-reinforced by structural inequities in housing, education, and policing continue to shape willingness to engage in clinical research.

Given these patterns, it is reasonable to expect a negative relationship between exposure to fatal police shootings and individuals’ trust in institutions, including research institutions.

### Conceptualizing Trust

While institutional trust has been discussed throughout, it is important to clarify the distinctions between related constructs. Trust is a multidimensional concept often centered on perceived risk in a relationship at either the individual or organizational level [21]. Although the terms *mistrust* and *distrust* are often used interchangeably in research [27], Marsh and Dibben’s [28] distinguish mistrust-the belief that the trustee will not act in the truster’s best interest- from distrust, which reflects an expectation of harm [28]. We believe using the appropriate term is necessary for researchers to understand and address barriers to participation in clinical research. Durant et al. [29], for example, assessed societal distrust using items such as “people like you will be used as guinea pigs without permission” and interpersonal distrust by items such as “belief that your doctor would not ask you to participate in harmful medical research.” They found that African American/Black participants expressed significantly greater societal distrust than White participants, even when levels of interpersonal distrust were similar. Such findings underscore the need to differentiate between interpersonal relationships and broader systemic trust developing inclusive research strategies.

### Social Vulnerability

Social vulnerability offers insight into how societal actions impact how we experience hazardous events differently. Individuals in disadvantaged neighborhoods tend to live in isolated environments with limited growth opportunities. A community’s capacity to avert human suffering and financial loss during a disaster may depend on how much particular social factors, such as high poverty, a low percentage of automobile access, or congested houses, are present there. These elements characterize the social vulnerability of a community [30]. Social capital, beliefs, customs, physical population limitations, the type and density of infrastructure and lifelines, the built environment’s characteristics (building stock and age, for example), limited access to resources and political power, and physical limitations all contribute to social vulnerability [31]. Social and economic characteristics of a neighborhood have been shown to impact health outcomes such as cognitive health [32]. Furthermore, police tend to assign higher situational risk factors to communities with greater economic hardship and racial conflict; and these counties are more likely to experience fatal police force [33].

To objectively assess community resources and identify areas most in need, the Agency for Toxic Substances and Disease Registry’s Geospatial Research, Analysis, and Services Program collaborated with the Centers for Disease Control and Prevention, National Center for Environmental Health, and Office of Terrorism Preparedness and Emergency Response (OTPER) to create a social vulnerability index (SVI) that would help other-funded state partners during all stages of the disaster cycle. This index enables disaster management professionals from state, local, and tribal governments to locate their most susceptible populations. Building on studies that view vulnerability as a societal condition or a gauge of population groups’ ability to withstand adversity [31], the SVI provides an objective framework for understanding and addressing the diverse needs of communities.

### Research Aims

**Fig. 2:**
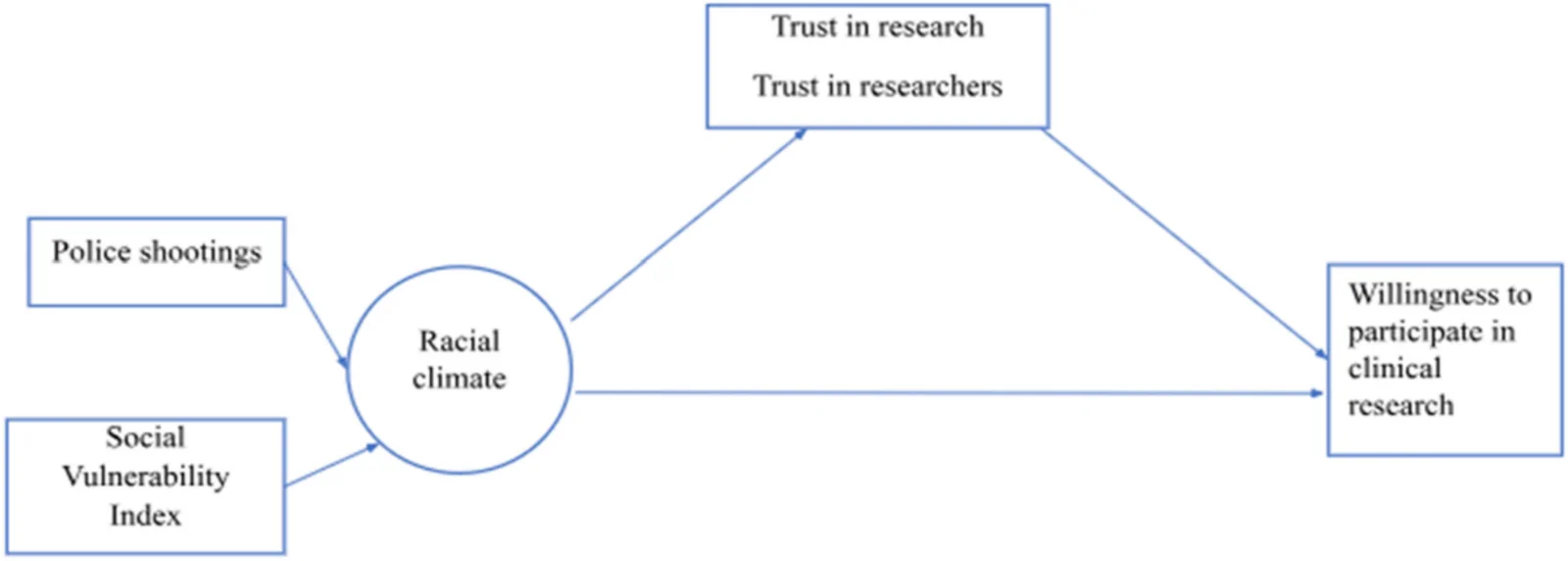
The conceptual model of research aims

Using the socio-ecological model [34] as a framework, this project examined the relationship between the racial climate of the neighborhood and adults’ willingness to participate in clinical research. Specifically, this study assessed whether the number of police shootings and the level of social vulnerability are negatively associated with individuals’ willingness to participate in clinical research. We further explored the role of trust in the relationship between police shootings and individuals’ willingness to participate in clinical research (Fig. 2).

We hypothesized that individuals residing in areas with higher social vulnerability index would be less likely to be willing to in participate in clinical research. Additionally, we hypothesized that African American/Black individuals who have experienced police shootings in their communities—defined by county and zip code—are less likely to be willing to participate in clinical research than White participants. We further expected that trust in research or researchers may explain this relationship.

## Methods

We used data from HealthStreet, a registry based in University of Florida community engagement program that utilizes community health workers (CHW) to assess the health concerns and conditions of the community [35]. HealthStreet CHWs approach community members in different locations such as community service agencies, health fairs, faith-based organizations, recreational parks, grocery stores, retail centers, and laundromats. The HealthStreet methodology has been described previously [35, 36].

### Participants

Participants were community members who were interviewed from November 2011 to May 2024. All participants signed the consent form, approved by the University of Florida Institutional Review Board. The interviews were about 30 min and obtained information about individuals’ health conditions, health concerns, sociodemographic factors, and willingness to participate in various types of clinical research studies. While HealthStreet CHWS data is available from late 2011 to mid-2024, due to data limitations of the Fatal Encounters dataset, the analysis in this paper will only use data from November 2011 to December 2021.

### Measures

#### Dependent Variables

Participants were asked whether they had participated in clinical research before and were willing to participate in different types of health studies. Specifically, they were asked whether they would volunteer for a clinical research study that (a) “Only asked questions about your health,” (b) “If researchers wanted to see your medical records,” (c) “if you had to give a blood sample,” (d) “If you were asked to give a sample for genetic studies,” (e) “If you might have to take medicine,” (f) “If you were asked to stay overnight in a hospital or clinic,” (g) “If you might have to use medical equipment.” Participants were also asked whether they would participate in a study without compensation (yes or no response).

#### Independent Variables

##### Social Vulnerability Index (SVI)

SVI was constructed based on census tracts, which are small subdivisions of counties commonly used for planning and policy in public health and government [30, 37]. The index contains four domains: socioeconomic status, household composition and disability, racial and ethnic minoritized status and language, and housing and transportation. The supplemental file Part A includes the variables in each SVI theme. We used the aggregated overall vulnerability for our analyses. This study used the last published SVI rankings in 2020.

##### Fatal Encounters

Fatal police shootings described in this paper relies on data from survey(s) administered by the National Officer-Involved Homicide Database, which is maintained by the Center for Economic and Social Research (CESR) at the University of Southern California. The Fatal Encounters project began collecting police shooting data at the beginning of the year 2000. The latest fatal police shooting was recorded in December 2021. Comer and Ingram [38] previously analyzed the similarities and differences between Fatal Encounters data and two other open-source datasets, Washington Post and *Mapping Police Violence.* Their findings showed a strong linear correlation among all three datasets. For the purpose of this study, we excluded cases that were identified as suicide from our analyses.

##### Trust in Research (HealthStreet Data)

Participants were asked on a scale of 1 to 10, how much they trust research (where 1 is “not at all” and 10 is “completely”).

##### Trust in Researchers (HealthStreet Data)

Participants were asked on a scale of 1 to 10, how much they trust researchers (where 1 is “not at all” and 10 is “completely”).

### Statistical Analysis

We merged HealthStreet, Fatal Encounters, and social vulnerability index datasets by County. The analytical dataset included observations from 2011 to 2021. Although HealthStreet CHWS data is available from late 2011 to mid-2024, this paper only used data from November 2011 to December 2021 with 9219 HealthStreet participants.

Descriptive statistics were calculated for participants’ characteristics. We tested for the effect of time and visualized willingness to participate in different clinical research scenarios over the years. A chi-square test of independence was performed to examine the relationship between race and willingness to participate in clinical research. Since the majority of participants were African American/Black, and police shootings impact African American/Black individuals disproportionately, we compared their willingness to participate with White participants. We examined the relationship between our independent variables: social vulnerability index (SVI) and fatal police shootings using correlation coefficient. Multiple logistic regression models were used for continuous independent and categorical dependent variables to predict willingness to participate in clinical research based on the number of fatal police shootings and SVI among African American/Black and White participants. Models included age, gender, and education as covariates. Race was coded as a binary variable with White as the reference group. The odds ratio (OR = e^b^) was calculated. Given the multiple logistic regression models conducted, a Bonferroni correction was applied to the *p*-values. Mediation analyses were used to determine whether trust mediated the relationship between fatal police shootings and willingness to participate in clinical research. Total effect, indirect effect (Average Causal Mediation Effect, ACME) and direct effect (Average Direct eEffect, ADE) were calculated. ACME estimates the portion of the effect of fatal police shootings on willingness to participate in clinical research that operates through trust in research and trust in researchers. Covariates included age, gender, and education. RStudio was used for all analyses. *p*-values less than 0.05 were considered to be statistically significant.

We then visualized police shootings at a more granular level by plotting the number of police shootings in each zip code within a County and participants’ willingness to participate in clinical research. Since we had more data from 35 zip codes, with 143 police shootings within Duval County versus 29 zip codes and 22 fatal shootings in Alachua, we focused solely on Duval County zip codes for this visualization. We calculated the weighted percentage of willingness to participate based on the number of participants in each zip code. We visualized zip codes with a high number of shootings (5 or more) to zip codes with a low number of police shootings (4 or less) from the years 2011–2021 (*N* = 2357). PowerBI was used to create visuals.

## Results

### HealthStreet Participants

The average age of HealthStreet participants was 43.01 years (SD = 16.32). Average education was 13.04 years (SD = 2.35). The gender distribution was predominantly female (63.6%), followed by male (36.2%), with a small percentage identifying as transgender, nonbinary, or other (0.2%). Most participants (56.2%) identified themselves as Black/African American, followed by 36.3% White, 4.7% Other, 1.6% Asian, and 1.3% American Indian/Alaskan Native. Comparing HealthStreet sample to the Florida population, 15.1% of the state population identify as Black/African American individuals, 51.53% as non-Hispanic White, and 26.4% as Hispanic/Latino. Of 5174 Black/African American participants, (98.4%) were non-Hispanic Black/African American. Out of 3340 White participants, 10.3% identified as Hispanic. The majority of participants were not currently married (not married/other = 72.2%). The top participants’ neighborhood concerns were “no concerns” (22.5%), “safety or security” (11.6%), “crime” (8.2%), and “drugs” (7.7%). Table 1 illustrates the characteristics of the sample.

**Table 1:** Demographic characteristics of African American/Black and White participants, *N* = 9219

	African American/Black participants *N* = 5174			White participants *N* = 3340			All participants *N* = 9219		
Variable	*M* (*SD*)	*Median*	*N*	*M* (*SD*)	*Median*	*N*	*M* (*SD*)	*Median*	*N*
Age	42.17 (15.31)	42		45.45 (17.46)	46		43.01 (16.32)	43	
Education	12.52 (2.12)	12		13.75 (2.81)	13		13.04 (2.53)	12	
Gender									
Female			3363 (62.3%)			2201 (65.9%)			5862 (63.6%)
Male			2023 (37.6%)			1124 (33.7%)			3334 (36.2%)
Transgender_1_			6 (0.1%)			13 (0.4%)			23 (0.2%)
Employed			1976 (38.4%)			1296 (39%)			3570 (38.7%)
Unemployed			3173 (61.6%)			2030 (61%)			5605 (60.8%)
Household size	2.52 (2.43)	2		2.12 (1.39)	2		2.37 (1.49)	2	
Marital status									
Married			1158 (22.4%)			1192 (35.7%)			2557 (27.8%)
Not married			2754 (53.3%)			1162 (34.8%)			4276 (46.5%)
Other_2_			1253 (24.3%)			981 (29.4%)			2369 (25.7%)
Veteran			386 (7.5%)			318 (9.5%)			763 (8.3%)
Ethnicity									
Hispanic			84 (1.6%)			341 (10.3%)			829 (7.4%)
Non-Hispanic			5065 (98.4%)			2979 (89.7%)			9180 (92.6%)

*Responses with age less than 18 were excluded from the summary statistics

**Responses with household size greater than ten were excluded from summary statistics

^1^ Transgender or nonbinary

^2^ Widowed, divorced, or separated

Participants were recruited from different Florida counties with the top three counties being Alachua (63.7%), Duval (18.2%), and Putnam (6.2%). The supplemental file provides information on the number of shootings for each county. Fatal police shootings in Florida are graphed in the supplemental file.

### Willingness to Participate Over Time

**Fig. 3:**
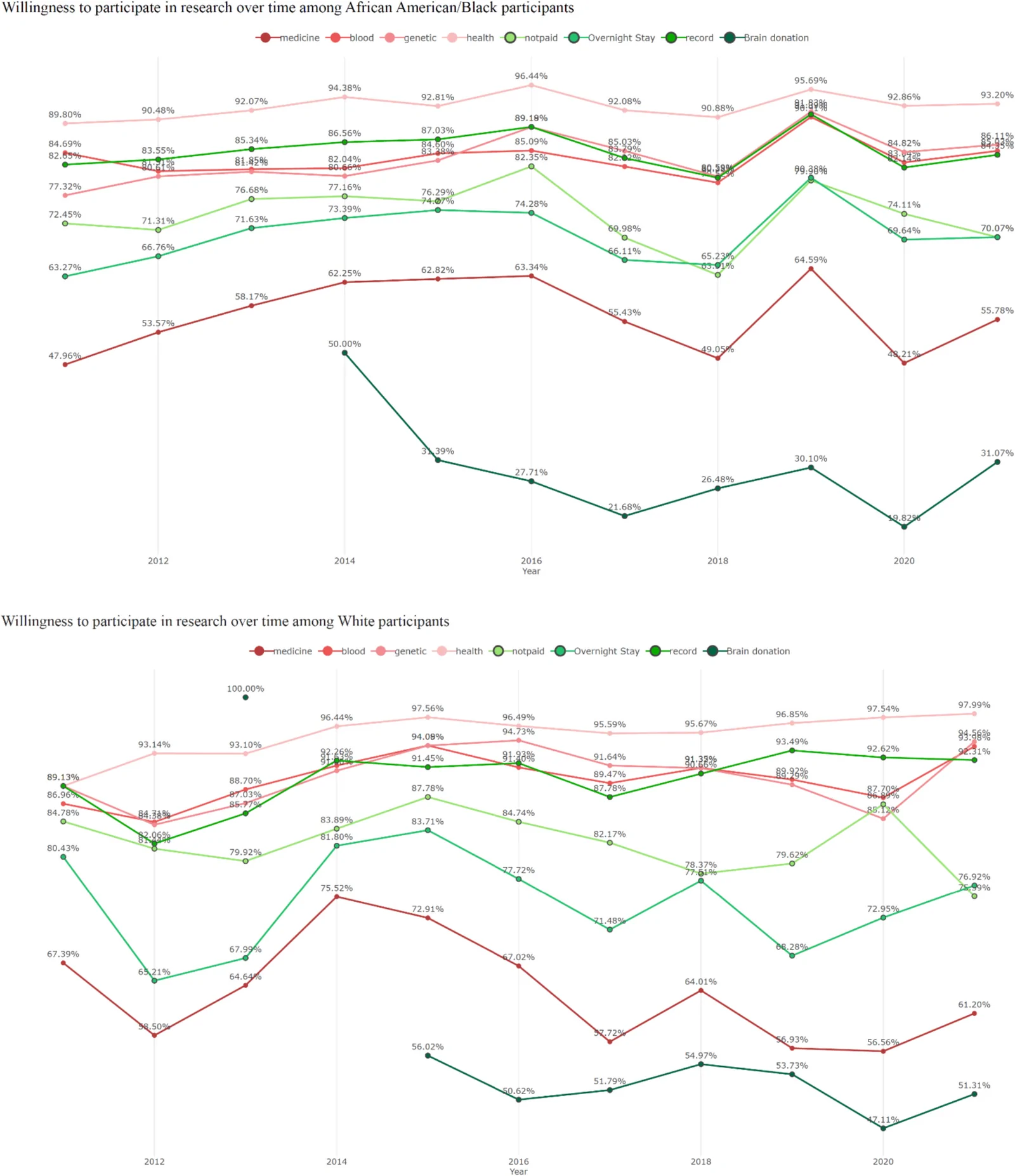
Willingness to participate in clinical research from 2011 to 2021

Overall, there was a slight increase in willingness to participate from 2011 to 2021. Willingness to take medicine, brain donation, and overnight stay had the lowest likelihood among both groups (Fig. 3). We observed that the trend among African American/Black and White participants differed over the years. For instance, between 2016 and 2018, African American/Black participants were less likely to participate in clinical research; however, willingness to participate among White participants decreased in the year 2017 but increased in 2018. A similar decline in willingness to participate was not observed among White participants compared to African American/Black willingness in 2020**.** Survey inventory related to brain donation started later in 2014 and after the initial year of implementation, willingness to donate brain remained around 20 to 30%.

### Willingness to Participate and Race

**Fig. 4:**
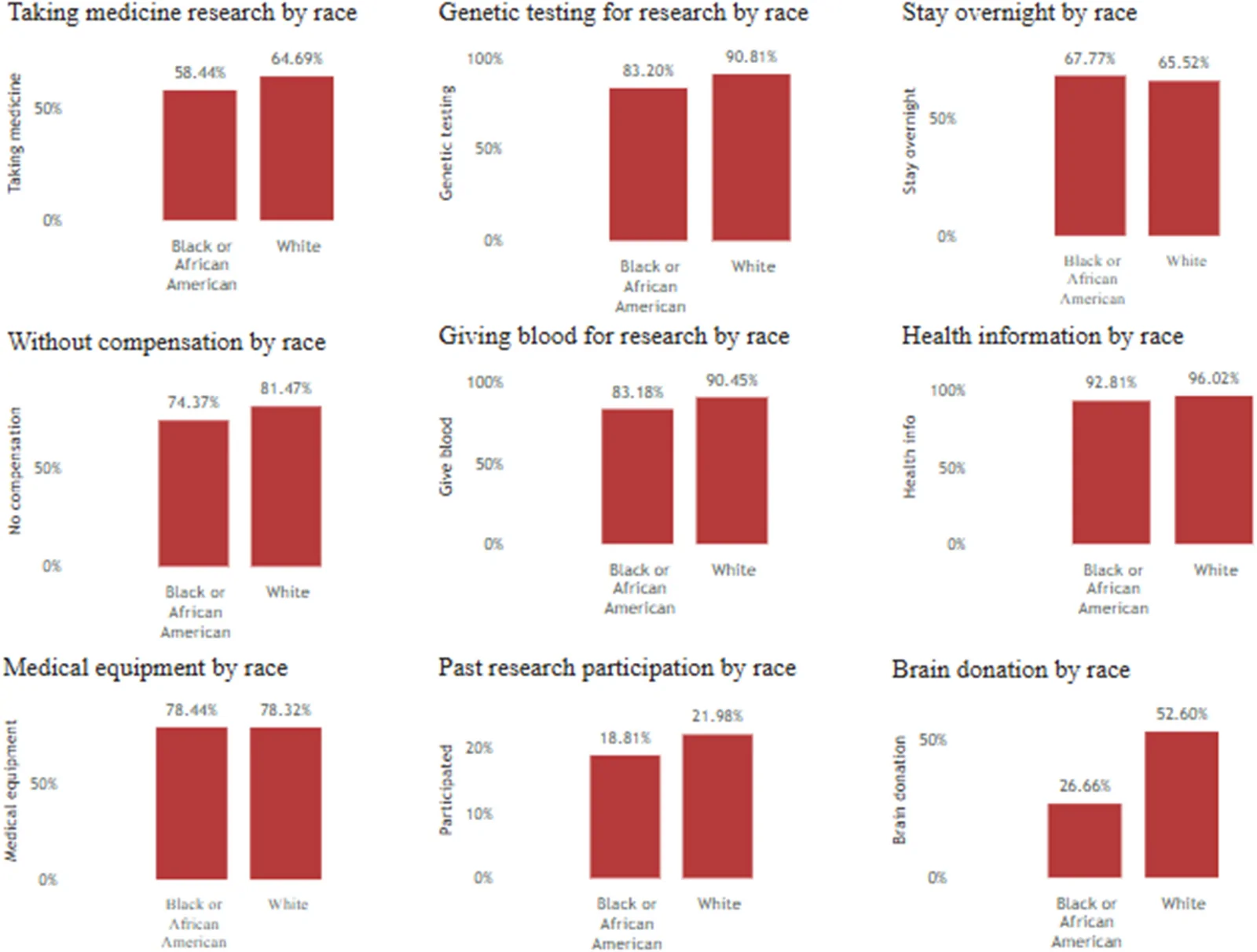
Differences between the likelihood of willingness to participate in clinical research among African American/Black and White participants. Clinical research scenarios: taking medicine for research, providing samples for genetic testing, staying overnight, participating in research without being compensated, giving blood, providing health information, using medical equipment, reporting past research participation and brain donation

A chi-square test of independence was performed to examine the relationship between race and willingness to participate in clinical research. Figure 4 demonstrates the calculated percentage of the likelihood of willingness to participate in different clinical research scenarios by race. Overall, African American/Black participants were less willing to participate in clinical research that required to take medicine (58.44% vs. White 64.69%, *X*^2^(1) = 59.79, *p* < 0.001), genetic testing (83.20% vs. White 90.81%, *X*^2^(1) = 151.52, *p* < 0.001), stay overnight (65.52% vs White 67.77%, *X*^2^(1) = 19.83, *p* < 0.001), not getting paid/without compensation (74.37% vs. White 81.47%, *X*^2^(1) = 96.78, *p* < 0.001), give blood (83.18% vs White 90.45%, *X*^2^(1) = 132.6, *p* < 0.001), share health information (92.81% vs. White 96.02%, *X*^2^(1) = 52.90, *p* < 0.001), or past research participation (18.81% vs. White 21.98%, *X*^2^(1) = 33.33, *p* < 0.001).

### Social Vulnerability Index and Willingness to Participate in Clinical Research

Correlation analysis showed that a higher number of police shootings was associated with a higher overall social vulnerability index (*r* = 0.27, *p* < 0.05). Multiple logistic regression models were run with overall social vulnerability index, and race as predictors, and willingness to participate in clinical research as dependent variables. Age, education, and gender were set as covariates. Results showed that African American/Black participants reported less willingness to participate in clinical research than White participants in all clinical research scenarios (Table 2). After the Bonferroni adjustment, the overall vulnerability was associated with a lower likelihood of willingness to participate in the majority of clinical research scenarios (Table 2).

**Table 2:** Social vulnerability index (SVI) and willingness to participate in clinical research among African American/Black compared to White participants. Age, gender, and education were set as covariates

Variable	African American/Black					Overall SVI					*Adjusted p*
	*B*	*SE*	*p*	CI	*OR*	*B*	*SE*	*p*	CI	*OR*	
Give blood	− 0.59	0.07	0.0000	[− 0.7272, − 0.4528]	0.554	− 5.11	1.27	0.0000	[− 7.5992, − 2.621]	0.006	0.0128
Take medicine	− 0.26	0.05	0.0000	[− 0.3580, − 0.1620]	0.771	− 5.29	0.91	0.0001	[− 7.0736, − 3.5064]	0.005	0.0157
Genetic testing	− 0.64	0.08	0.0000	[− 0.7968, − 0.4832]	0.527	− 6.15	1.27	0.0000	[− 8.6392, − 3.6608]	0.002	0.0117
Use medical equipment	− 0.46	0.08	0.0000	[− 0.6168, − 0.3032]	0.631	− 9.28	1.34	0.0000	[− 11.9064, − 6.6536]	0.000	0.0012
Without compensation	− 0.43	0.05	0.0000	[− 0.5280, − 0.3320]	0.649	− 9.42	1.02	0.0001	[− 11.4192, − 7.4208]	0.000	0.0924
Health information	− 6.79	1.2	0.0000	[− 9.1420, − 4.4380]	0.001	− 10.35	1.84	0.0000	[− 13.9564, − 6.7436]	0.000	0.0921
Health records	− 6.08	0.08	0.0000	[− 6.2368, − 5.9232]	0.002	− 6.26	1.31	0.0000	[− 8.8276, − 3.6924]	0.002	0.0941
Overnight stay	− 0.23	0.06	0.0001	[− 0.3476, − 0.1124]	0.793	− 7.45	1.04	0.0000	[− 9.4884, − 5.4116]	0.001	0.0003
Brain donation	− 1.34	0.06	0.0000	[− 1.4576, − 1.2224]	0.263	− 7.27	1.1	0.0000	[− 9.4260, − 5.1140]	0.001	0.0296

### Police Shooting

We ran multiple logistic regression with the number of police shootings as a predictor and willingness to participate in clinical research as dependent variables. Age, gender, and education were set as covariates. Results showed that a higher number of police shootings was significantly associated with a lower willingness to participate in clinical research that required giving blood (*b* =  − 0.139, se = 0.004, *p* < 0.01, OR = 0.870), take medicine (*b* =  − 0.124, se = 0.055, *p* < 0.05, OR = 0.884), providing samples for genetic studies (*b* =  − 0.001, se = 0.000, *p* < 0.05, OR = 0.999), use medical equipment (*b* =  − 0.021, se = 0.001, *p* < 0.001, OR = 0.979), and past research participation (*b* =  − 0.033, se = 0.000*, p* < 0.001, OR = 0.968). Fatal police shooting was associated with a slightly higher willingness to participate without compensation (*b* = 0.025, se = 0.007, *p* = 0.001, OR = 1.025). Table 3 demonstrates results from the number of police shootings as a predictor of willingness to participate in clinical research. To account for multiple testings, a Bonferroni correction was applied to the *p*-values. After adjustment, the relationship between police shootings and giving blood was no longer significant (adjusted *p* > 0.05), while all other associations remained significant.

**Table 3:** Multiple logistic regression models with the number of police shootings as a predictor and willingness to participate in clinical research as dependent variables among African American/Black and White participants. Age, gender, and education were set as covariates

Willingness to participate variables	*B*	*SE*	*p*	CI	*OR*	*Adjusted p*
Give blood	− 0.139	0.004	0.0053	[− 0.1478, − 0.1302]	0.870	0.7312
Take medicine	− 0.124	0.055	0.0288	[− 0.2328, − 0.0152]	0.884	0.0022
Genetic sample	− 0.001	0.000	0.0369	[− 0.001, − 0.001]	0.999	0.0062
Use medical equipment	− 0.021	0.001	0.0000	[− 0.0229, − 0.019]	0.979	0.0471
Without compensation	0.025	0.007	0.0011	[− 0.0113, − 0.0387]	1.025	0.0022
Health information	0.001	0.000	0.8751	[− 0.001, 0.001]	1.011	0.4259
Health records	0.014	0.009	0.1375	[− 0.0036, 0.0316]	1.014	0.4213
Overnight stay	0.010	0.007	0.1759	[− 0.0037, 0.0237]	1.010	0.2688
Brain donation	0.034	0.91	0.706	[− 1.7456, 1.8136]	1.035	0.7802

Among Florida counties, we prioritized Alachua and Duval counties since they had the highest number of participants and fatal police shootings (see Supplemental file). In Alachua, African American/Black participants (*b* =  − 0.45, se = 0.06, *p* < 0.001, OR = 0.64) were less likely to take medication (*b* =  − 0.65, se = 0.2, *p* < 0.01, OR = 0.52) and stay overnight (*b* =  − 0.70, se = 0.22, *p* < 0.01, OR = 0.49) for clinical research compared to White participants. In Duval County, African American/Black participants (*b* =  − 0.68, se = 0.14, *p* < 0.001, OR = 0.51) were significantly less likely to take medication (*b* =  − 0.15, se = 0.05*, p* < 0.01, OR = 0.86), use medical equipment (*b* =  − 0.13, se = 0.06, *p* < 0.05, OR = 0.88), stay overnight (*b* =  − 0.12, se = 0.05, *p* < 0.05, OR = 0.88), or report past research participation (*b* =  − 2.23, se = 1.5, *p* < 0.001, OR = 0.11) compared to White participants.

**Fig. 5:**
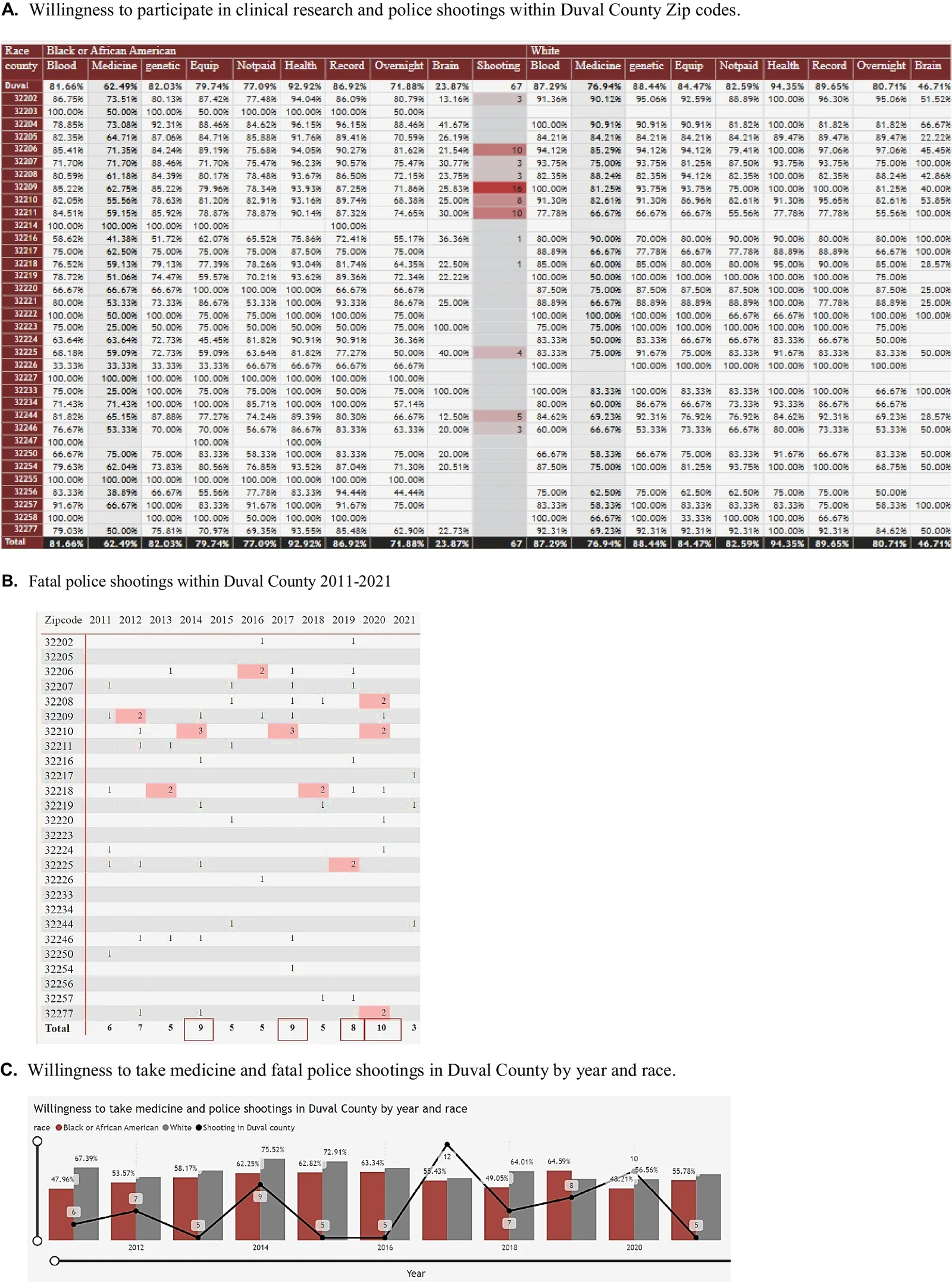
**A** Willingness to participate in clinical research that requires giving blood, taking medication, and genetic testing, using medical equipment, without compensation, sharing health information, sharing health records, staying overnight and brain donation by race in Duval County zip between 2011 and 2021 (*N* = 2357). Column *Shootings* represents the total number of fatal police shootings in each zip code. **B** The number of fatal police shootings in Duval County zip codes between 2011 and 2021. **C** Weighted percentage of willingness to take medicine based on the number of participants in each zip code by race in Duval County in 2011–2021

Next, we visualized police shootings and clinical research perceptions by Zip code in Duval County (Due to its large number of study participants and fatal police shootings). The difference between willingness to take medication was more pronounced among African American/Black compared to White participants (see Fig. 5), specifically in zip codes with higher fatal police shootings (see Fig. 5A). We next visualized the number of police shootings across the years (Fig. 5B) and with willingness to take medication (Fig. 5C). The highest number of shootings within Duval County occurred in 2014 (nine shootings), 2019 (eight shootings), and 2020 (10 shootings). The visualization suggested a lower likelihood of willingness to take medication in years (e.g., 2014 & 2020) with a higher number of police shootings among African American/Black individuals than White individuals.

### Trust

Independent *t*-test analyses showed that on average, White participants (*M* = 8.53, SD = 8.52, median = 8) report more *trust in researchers* compared to African American/Black (*M* = 7.75, SD = 8.04, median = 7) participants (*t* =  − 2.67, df = 7876, *p* < 0.01). Similarly, White participants (*M* = 8.53, SD = 8.99, median = 8) tend to have *trust in research* more than African American/Black (*M* = 7.75, SD = 9.69, median = 7) participants (*t* =  − 4.15, df = 7893, *p* < 0.001). We then examined whether trust served as a mediator between fatal police shootings and participants’ willingness to participate in clinical research. Results showed that police shooting was significantly associated with lower trust in research (*b* =  − 0.11, se = 0.03, *p* < 0.01) and researchers (*b* =  − 0.15, se = 0.04, *p* < 0.001). Both trust in research (ACME =  − 0.0004, *p* < 0.01) and trust in researchers (ACME =  − 0.0003, *p* < 0.01) mediated the relationship between police shootings and lower willingness to take medication for clinical research. The average direct effect of police shootings and willingness to take medicine was no longer significant in the mediation analyses (ADE = 0.003, *p* = 0.102). Results showed 15.3% of the total effect was mediated by trust in research and 12.4% of the total effect was mediated by trust in researchers.

## Discussion

The contribution of racial climate to clinical research participation among African American/Black communities is poorly understood. Previous research suggests that African American/Black individuals are more likely to experience police brutality [17–19] than White individuals. As researchers, it is crucial to consider a broader context when approaching underrepresented community members with high levels of distrust in institutions. In this study, we used fatal police shootings as a proxy for racial climate, and we aimed to understand the role of trust in the individuals’ perceived willingness to participate in clinical research. Our findings indicate that individuals are less likely to be willing to participate in clinical research where there is a high number of fatal police shootings. We also found that African American/Black individuals are less likely to trust research and researchers compared to White individuals. In addition, low trust in research and researchers explains the relationship between fatal police shootings and individuals’ being willing to take medicine for clinical research.

Although exposure to fatal police shootings was not significantly associated with individuals’ willingness to donate brain for Alzheimer’s clinical research, we found that African American/Black participants were still less likely than White participants to express willingness to donate their brain. Previous research has shown that apprehension towards brain donation is influenced by factors, such as religious/cultural beliefs, distrust in clinical research, family influence, or limited familiarity with the organ donation process [39]. These findings highlight the need for greater community engagement and culturally responsive education efforts to raise awareness and build trust around brain donation with African American/Black communities.

When assessing individual zip codes within Duval County, we found a difference in willingness to take medicine for clinical research between areas that had higher police shootings and those that had no or lower number of police shootings. Our findings supported our hypothesis that being in neighborhoods that expose individuals to experiencing police shootings can negatively impact their willingness to participate in clinical research. We believe that lower trust in research institutions mediates this relationship. This distrust, as shown in past research, is not without foundation. Historical injustices—most notably the Tuskegee Syphilis Study—have profoundly shaped African American communities’ relationship with medical research and healthcare institutions [22–26]. The echoes of such exploitation, along with current inequities like police brutality, contribute to a climate of justified skepticism.

In addition to police brutality, African American/Black individuals are impacted by other forms of structural racism. We found that African American/Black individuals were more likely to live in areas with higher social vulnerability index, and higher overall social vulnerability was negatively associated with individuals’ willingness to participate in clinical research. This may suggest that factors such as low socioeconomic status, lack of healthcare access, and lack of housing and transportation create more barriers for individuals to participate in clinical research. These challenges could result in African American/Black individuals being underrepresented in clinical research samples, potentially leading to biased results and less generalizable findings. Addressing these barriers is crucial for ensuring equitable participation. These findings can inform future recruitment methodologies, guiding the development of more inclusive strategies that accommodate the diverse needs of all potential participants.

The findings from this study should be considered with limitations. We used existing datasets captured mainly in North Central Florida. Perceptions of clinical research, participants’ demographics, and police shootings are limited to one state. Future research should examine the relationship between fatal police shootings and other forms of structural racism and clinical research perceptions in a more systematic approach to understand how mechanisms of structural racism erode trust in institutions and, consequently, individuals’ willingness to participate in clinical research. Moreover, given that the HealthStreet registry employs a community-based recruitment approach, leveraging CHWs in familiar and trusted settings, we suspect participants’ willingness to participate in clinical research and their trust in research and researchers is higher than in settings without community-engaged research practices. This study found a relatively small temporal difference in willingness to participate. These differences may be partially attributable to age disparities between racial groups, as White participants were, on average, 3 years older than African American/Black participants. Given the association between older age and increased health concerns, this may influence willingness to participate independent of race. Additionally, the sample’s mean age of 43 years is younger than typical clinical research cohorts, potentially limiting the generalizability of findings to populations at greater risk for age-related health outcomes. In addition, this study focused on clinical research perceptions and did not assess actual enrollment. Future research should explore how the relationship between structural racism and willingness to participate translates into the effect of structural racism on clinical research enrollment.

Data collection on clinical research perceptions is critical for understanding and addressing barriers to research participation, including racial injustice, socioeconomic inequities, and distrust in research institutions. By analyzing perceptions across these dimensions, we can identify strategies to improve inclusivity and equity in clinical research. These strategies can then be evaluated using enrollment rates. Beyond researchers, our findings can benefit a wide range of audiences, including students, funders, and policymakers interested in learning more about how to address the needs of the communities and reduce the erosion of trust in institutions among the racial and ethnic minoritized communities.

## Supplementary Information

Below is the link to the electronic supplementary material.

Supplementary file 1 (PDF 1.10 MB)

## Data Availability

Fatal Police Shootings data is available on Fatal Encounters: https://fatalencounters.org/ Healthstreet data is captured and maintained by the University of Florida community engagement program. For more information, visit healthstreet.program. Social Vulnerability Index (SVI) is available on CDC website: https://www.atsdr.cdc.gov/place-health/php/svi/index.html.
