# Supplementary material for: Racial Climate on Willingness to Participate in Clinical Research
Source: J Racial Ethn Health Disparities. 2025 Jul 7;13(5):3723–36. doi: 10.1007/s40615-025-02530-6 (PMC13546346; doi:10.1007/s40615-025-02530-6)

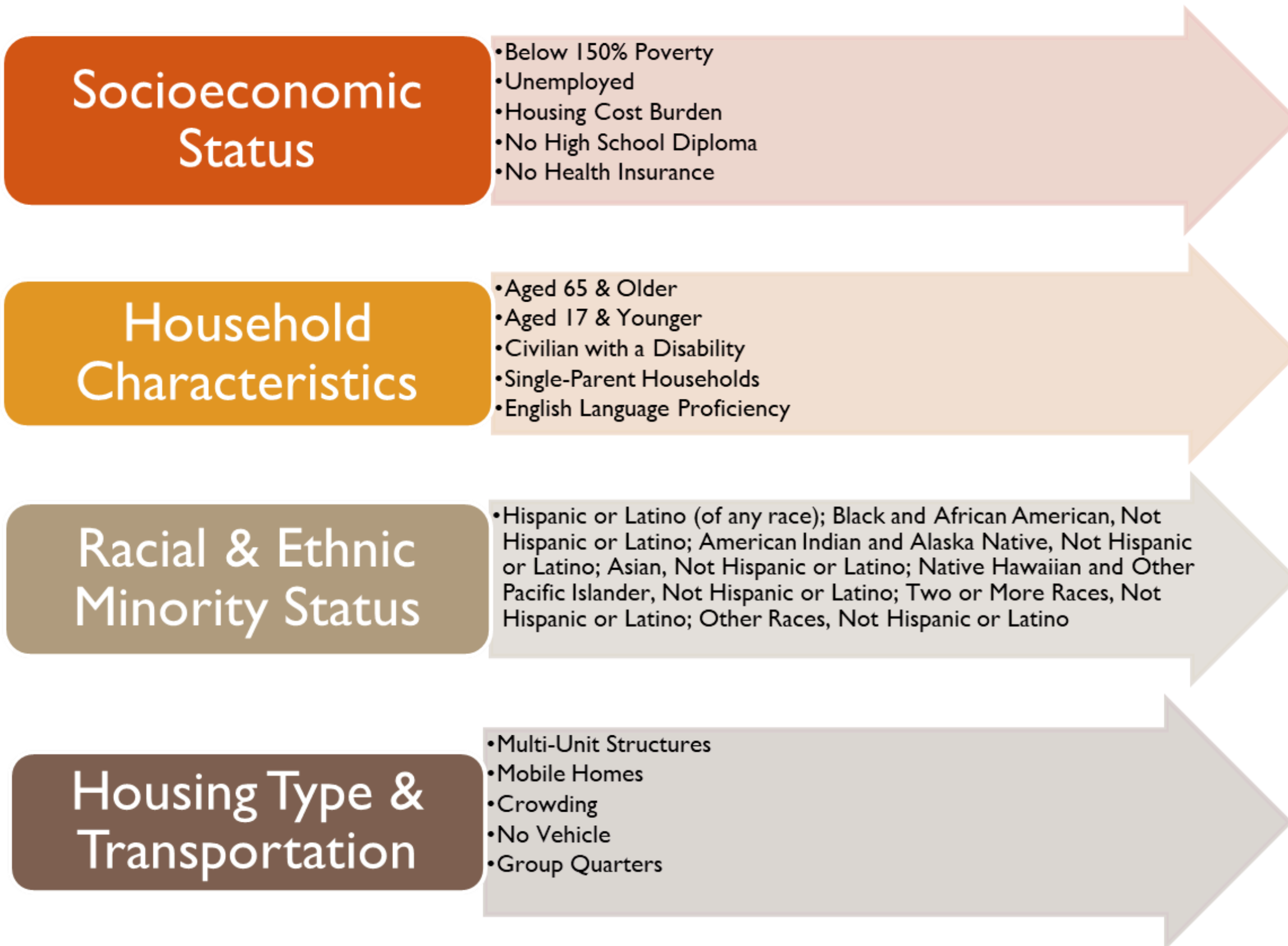

A. Social Vulnerability Index (SVI)

B. The number of fatal police shootings for each county

County	Police shootings
Alachua	22
**Duval**	**143**
Putnam	14
Union	2
Marion	25
Levy	2
Bradford	4
Columbia	9
Miami-Dade	177
Clay	15
Calhoun	
Gadsden	4

Gilchrist	2
St. Johns	23
Orange	72
Hillsborough	62
Suwannee	4
Palm Beach	48
Broward	57
Baker	4
Leon	23
Brevard	29
Hamilton	3
Volusia	39
Bay	16
Dixie	3
Lake	15
Pinellas	40
Seminole	15
Sumter	6
Citrus	9
Nassau	13
Osceola	5
Sarasota	11
Polk	13
Taylor	1
Lee	11
Flagler	4
Manatee	10
Pasco	12
Walton	
Collier	1
Escambia	15
Indian River	4
Jackson	2
Santa Rosa	3
Tarrant	
Washington	2
Williamson	
Charlotte	2
DeSoto	1
District of Columbia	

Harris	
Highlands	1
Kings	
Lafayette	
Lowndes	
Martin	5
Monroe	4
Okaloosa	1
Richmond	
Baltimore	
Bibb	
Brooke	
Chesterfield	
Chicot	
Colquitt	
Coshocton	
DeKalb	
Fairfax	
Fayette	
Greene	
Greenville	
Gulf	3
Gwinnett	
Hardee	
Hernando	4
Hidalgo	
Ingham	
Laurens	
Liberty	
Madison	1
Montgomery	
New York	
Oklahoma	
Oneida	
Philadelphia	
Pickens	
Prince George's	
Shelby	
Solano	
St. Lucie	1
Suffolk	

Tehama	
Thomas	
Ventura	
Wakulla	
Williamsburg	
York	

C. Ratio of police shootings by county population in Florida

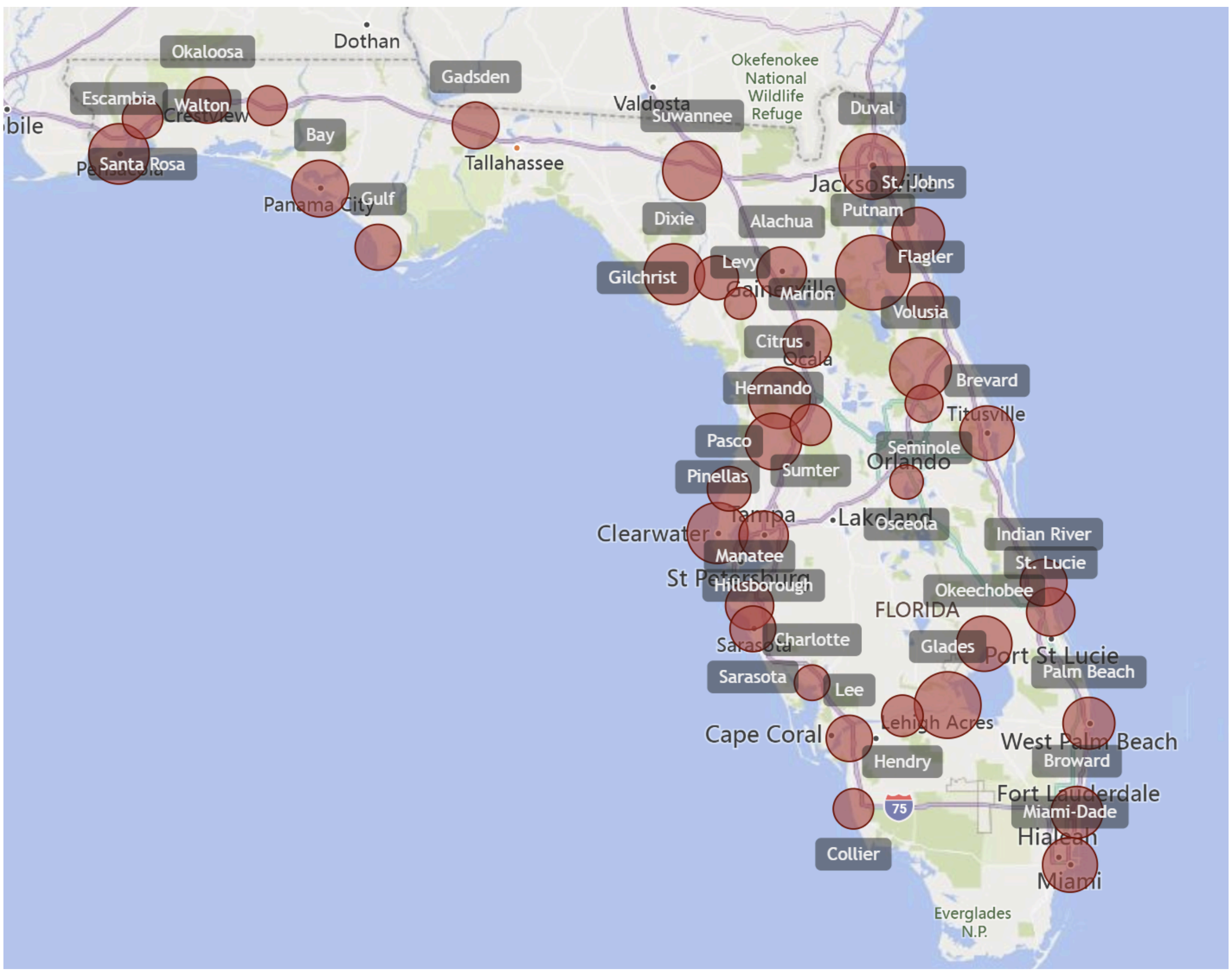

Supplement: Supplementary file 1 [file 40615_2025_2530_MOESM1_ESM.pdf]
